# Neonatal mortality by gestational age in days in infants born at term: A cohort study in Sao Paulo city, Brazil

**DOI:** 10.1371/journal.pone.0277833

**Published:** 2022-11-21

**Authors:** Marcel Reis Queiroz, Maria Elizangela Ramos Junqueira, Alejandra Andrea Roman Lay, Eliana de Aquino Bonilha, Mariane Furtado Borba, Célia Maria Castex Aly, Roberto Aparecido Moreira, Carmen Simone Grilo Diniz

**Affiliations:** 1 Health Department 3, Nove de Julho University, Sao Paulo, São Paulo, Brazil; 2 Gender and Evidence in Maternal Health (GEMAS), School of Public Health, University of São Paulo, São Paulo, Brazil; 3 Department of Life Sciences, Public Health Area, State University of Bahia, Salvador, Bahia, Brazil; 4 Subdepartment of Epidemiological Surveillance, Health Surveillance Board, Salvador Municipal Health Department, Salvador, Bahia, Brazil; 5 Faculty of Health Sciences, University of Tarapacá, Arica, Chile; 6 Municipal Health Department of Sao Paulo, Sao Paulo, São Paulo, Brazil; 7 São Camilo University, São Paulo, Brazil; 8 Department of Epidemiology, Faculty of Public Health, University of Sao Paulo, Sao Paulo, São Paulo, Brazil; 9 Department of Health, Life Cycles and Society, Faculty of Public Health, University of Sao Paulo, São Paulo, São Paulo, Brazil; University of Washington, UNITED STATES

## Abstract

Birth at term comprises a period with heterogeneous neonatal outcomes that tend to be worse for infants born earlier. However, few studies have analyzed this period, in which each day can make a difference. Therefore, we aim to assess neonatal mortality (NM) according to gestational age (GA) at birth measured in days in term liveborn infants born in 2012–2017 in São Paulo, the largest city in Latin America. This population-based cohort study assessed term liveborn infants followed until the end of the neonatal period. We analyzed 7 models for NM according to GA in days: crude NM adjusted for maternal and prenatal variables, NM additionally adjusted for type of birth and type of hospital, and adjusted NM stratified by type of birth (cesarean and vaginal) and by type of hospital (public and private). We included 440,119 live infants born at 259–293 days of gestation. The median GA at birth was 274 days. In all models, NM was higher for infants born early term, decreasing in infants born full term and rising again in infants born late term. In the unadjusted model, hazard ratios of NM changed daily, decreasing from 3.34 to 1.00 on day 278 and increasing again thereafter. In the stratified analysis according to type of hospital, being born in a public hospital was associated with a reduced risk of NM for infants born at 278–283 days of pregnancy. There was a decrease in GA related to obstetric interventions, especially cesarean sections, which increased NM. The loss of days of pregnancy was larger in private hospitals. Increasing the granularity of GA to days is feasible and has the potential to drive public policies. To the best of our knowledge, this is the first Brazilian study on GA in days using a national live births database.

## Introduction

Birth at term is defined as birth between 37–42 completed weeks of gestation (259 to 293 days). Babies born before 37 completed weeks (< 259 days) are classified as pre-term, while those born after 42 completed weeks (≥ 294 days) are post-term [1]. However, the term period is not as homogeneous as expected. Many studies found worse outcomes for infants born at 37–38 weeks of gestation compared to those born at 39–41 weeks, indicating risk variation in the period traditionally regarded as term [2, 3].

In a cohort study involving term births in the United States in 1995–2001, Zhang and Kramer [4] reported that mortality decreased with increasing gestational age (GA) from 0.66 deaths/1000 live births at 37 weeks to 0.33/1000 live births at 39 weeks and remained stable at 39–40 weeks. Wu et al. analyzed differences in outcomes of term infants according to GA in days in a Danish dataset from 1997–2004 and reported a sharp decrease in mortality for infants born after 280 days of gestation (40 weeks) [5].

Several Brazilian studies also found differences in outcomes of infants born at term. In a cohort study of live births (1982–2004) in a southern Brazilian city, Barros et al. [3] reported that infants born at 37–38 completed weeks of gestation had a higher risk of neonatal mortality (death between 0–27 days of life) and infant mortality (death between 0–365 days of life) than those born at 39–41 weeks. Another Brazilian study analyzed 18,652 live births in over 260 hospitals in 2012 and found a higher risk of NM in infants born at 37–38 weeks compared to those born at 39–40 weeks [6].

Several researchers have recommended that term births be subdivided into early term (37–38 6/7 weeks), full term (39–40 6/7 weeks), and late term (41–41 6/7 weeks). Some have also recommended that the period between 34–36 6/7 weeks be called late pre-term and that births occurring after 41 weeks be called post-term [7, 8]. According to Diniz et al. [9], it would be relevant to assess differences in GA in days, instead of weeks, to investigate factors associated with shorter pregnancies and their outcomes. Researchers have also proposed a new way of measuring GA in “potential pregnancy days lost” (PPDL), calculated by subtracting GA at birth in days from 280 days, to analyze factors associated with these lost days [9, 10]. The selection of 280 days (40 completed weeks) for this calculation is because this is the median GA of spontaneous deliveries in many studies and because adverse neonatal outcomes are less frequent after this point in time [5, 9].

The objective of this study was to assess neonatal mortality (0–27 days of life) according to GA in days in a cohort of liveborn infants born in 2012–2017 in the city of Sao Paulo, Brazil.

## Materials and methods

### Study design and participants

**Fig 1:**
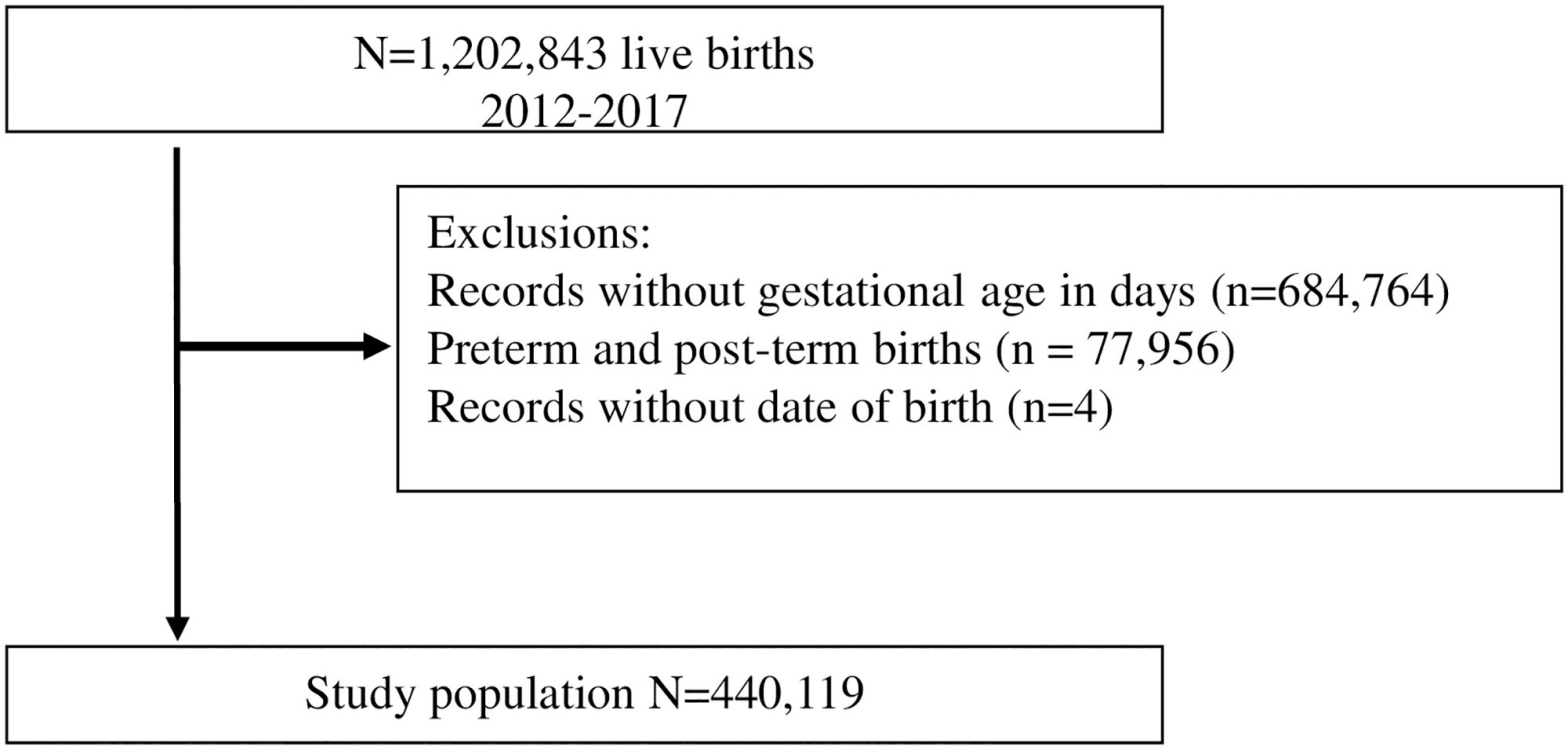
Study population.

This population-based cohort study analyzed live births between January 1, 2012, and December 31, 2017, in the city of São Paulo, Brazil, followed until the end of the neonatal period (28 days of life). According to the Brazilian Live Births Information System (SINASC), there were 1,202,843 live births during this period. In this study, we only included liveborn infants with information on GA in days who were born at term (between 259 and 293 days of pregnancy), resulting in 440,119 liveborn infants (Fig 1).

### Ethical aspects

This study is part of the "Potential pregnancy days lost (PPDL): An innovative gestational age measure for assessing maternal and child health interventions and outcomes” project (Bill and Melinda Gates Foundation, investment ID OPP1201939, and National Council for Scientific and Technological Development (CNPq), process number 443775/2018-4).

The project that was approved by the research ethics committee of São Paulo Faculty of Public Health (protocol number 98163018.2.0000.5421). Individual records were assigned a unique number to anonymize names, and we analyzed the data confidentially.

### Data sources

We obtained data from the Brazilian Live Births Information System (SINASC) and the Mortality Information System (SIM). Both databases are managed by the Epidemiological Coordination Unit (CEInfo) of the São Paulo Municipal Health Department. All variables in these databases are extracted from live birth certificates and death certificates.

To link the records of the two databases, we used three Visual Basic macros in Microsoft Excel and Microsoft Access. The first macro created graphic phonemes for the fields in text format. We used phonemes to identify similar first names of the mothers in the SINASC and SIM databases. The second macro cleaned first name fields by excluding both words that were not first names and numbers. The third macro created a similarity score between the fields that were transformed into graphic phonemes. We performed the following linkage steps: exclusion of newborn infants with implausible GA between 18–45 weeks; cleaning first names of the mothers in both databases (SINASC and SIM); elimination of unusual words (such as “report”, “unknown”, “stillborn”, “FF” and numbers); creation of “graphic phonetic phonemes” and “Soundex” phonetic algorithms for the first and last names of the mothers; generation of pairs through deterministic linkage between the number of the birth certificate and newborn sex in the two databases (SINASC and SIM); generation of the similarity score between the “graphic phonetic phonemes” and the “Soundex” algorithm pairs, plus exact match for date of birth and newborn sex; and verification of the pairs (cases with uncertainties were not considered pairs).

The process was made in a deterministic step using Microsoft Access, a second step probabilistic using Miscrosift Access and finaly a probabilistic step using Linkplus 2.0 from CDC, in order to confirm the pairs found by steps 1 and 2 and add any pairs not found in the previous steps. All three steps were manually reviewed by the researchers to ensure that the paired information was correct. Thes process produced a single merged database with all the relevant information on live births from 2012–2017 and all deaths of children up to 5 years of age in 2012–2018. This resulted in 13,347 pairs from the SINASC (2012–2017) and SIM (2012–2018) databases. We included only the pairs that had information on NM and GA at birth in days in this study. We used this merged database (from the SINASC and SIM databases) for all analyses.

### Outcome: Neonatal mortality

The main study outcome was all-cause neonatal mortality (NM), defined as the death of liveborn infants in the first 27 days of life from any cause.

### Independent variables

The main independent (predictor) variable was GA at birth in days (between 259 and 293 days) estimated on the basis of the first day of the last menstrual period (LMP) reported by the mother. The following maternal covariates were included as additional independent variables in the adjusted models: age (≤19, 20–34, ≥35 years), self-reported skin color (white or nonwhite), education (elementary, high school, university), living with a partner (yes or no), number of antenatal care visits (≥7, < 7), and parity (0, 1, ≥ 2 living children). We also included type of birth (cesarean or vaginal) and type of hospital (public or private) as associated variables.

### Statistical analyses

We used Cox regression models to calculate hazard ratios (HR) and 95% confidence intervals (95% CIs) to assess the association between GA in days and NM, adjusted for several covariates. We censored children who did not die by the last observation date (December 31, 2018). We modeled NM by GA in days using restricted cubic splines with five knots (262 days, 269 days, 274 days, 279 days and 288 days); 279 days was the reference knot because it was closer to 280 days, the central point of the full-term period. We first conducted a crude univariate analysis with GA in days (Model A). In Model B, we adjusted the results for maternal (age, skin color, education, living with a partner, parity) and gestational (number of antenatal care visits) variables. Model C was identical to the previous model with additional adjustments for type of birth (vaginal or cesarean) and type of hospital (private or public). We also conducted stratified analyses according to type of birth (Models D and E) and type of hospital (F and G). We used Stata version 15 (Stata Corp., College Station, Texas, USA) for all statistical analyses and to create the graphs.

We performed a sensitivity analysis for the sample using all 1,202,843 live births during the study period as a parameter but using gestational age in weeks. Crude and adjusted (for all independent variables) models were performed, with results similar to models made using only the sample with gestational age in days (details in S1 Table).

## Results

There were 1,202,843 live births during the study period. Among these, 684,764 only had data for GA in weeks, and 518,079 had data in weeks and days. After excluding women with GA between 130–258 days (n = 64,111), women with GA between 294–321 days (n = 13,845), and four women without information on the date of birth, we had a total of 440,119 eligible live births that were included in the study (Fig 1).

In all GA categories, 70.4% of women were 20–34 years of age, 59.3% had white skin, 86.7% had a high school or university education, 60.2% lived with a partner, 52% were giving birth for the first time, 65.3% had a cesarean section and 59.4% gave birth in private hospitals. More detailed information is provided in Table 1.

**Table 1:** Characteristics of the study population according to completed gestational weeks, municipality of São Paulo, Brazil, 2012–2018 (n = 440,119).

	Week 37 (259–265), n = 59.867		Week 38 (266–272), n = 131.608		Week 39 (273–279), n = 133.125		Week 40 (280–286), n = 82.387		Week 41 (287–293), n = 33.132	
	n	%	n	%	n	%	n	%	n	%
**Mother’s age**										
≤19	4,234	7.07	7,723	5.87	11,302	8.49	9,912	12.03	5,017	15.14
20–34	40,028	66.86	90,625	68.86	94,505	70.99	60,331	73.23	24,444	73.78
≥35	15,605	26.07	33,260	25.27	27,318	20.52	12,144	14.74	3,671	11.08
Skin color										
White	37,739	63.09	86,198	65.56	79,599	59.85	42,706	51.89	14,829	44.80
Nonwhite	22,074	36.91	45,279	34.44	53,389	40.15	39,588	48.11	18,274	55.20
**Mother’s education level**										
Elementary	6,891	11.53	12,539	9.54	17,072	12.84	14,273	17.35	7,326	22.15
High school	26,182	43.79	54,041	41.11	61,740	46.44	44,879	54.56	19,863	60.06
University	26,713	44.68	64,885	49.36	54,144	40.72	23,108	28.09	5,884	17.79
**Lives with partner**										
Yes	38,427	64.29	87,501	66.59	80,617	60.65	43,317	52.68	14,970	45.26
No	21,344	35.71	43,911	33.41	52,295	39.35	38,917	47.32	18,107	54.74
**Parity**										
0	29,638	49.55	66,496	50.57	70,044	52.66	45,077	54.77	17,588	53.13
1	19,746	33.01	45,636	34.70	42,978	32.31	24,769	30.10	9,987	30.17
2 or more	10,435	17.44	19,373	14.73	19,992	15.03	12,455	15.13	5,528	16.70
**Number of antenatal care visits**										
≤6	11,452	19.14	19,818	15.07	20,155	15.15	13,288	16.14	6,067	18.33
≥7	48,379	80.86	111,719	84.93	112,902	84.85	69,053	83.86	27,035	81.67
**Type of delivery**										
Vaginal	16,979	28.36	33,095	25.15	47,484	35.67	38,176	46.34	16,932	51.11
Cesarean	42,883	71.64	98,502	74.85	85,634	64.33	44,203	53.66	16,198	48.89
**Type of hospital**										
Private	40,712	68.37	96,556	73.73	80,615	60.95	35,158	43.06	8,507	25.92
Public	18,836	31.63	34,411	26.27	51,647	39.05	46,497	56.94	24,309	74.08

Out of the 2160 offspring deaths that occurred in 2012–2018, 797 took place in the first 27 days of life, 84.4% had at least one potential pregnancy day lost, and most were born by cesarean section (64%). See Table 2 for additional information on deaths.

**Table 2:** Neonatal mortality rate according to gestational age at birth in days, municipality of São Paulo, Brazil, 2012–2018.

Gestational age in days	Number of deliveries	Number of deaths	Neonatal mortality per 1000 live births
259–265	59.867	210	3.51
266–272	131.608	265	2.01
273–279	133.125	198	1.49
280–286	82.387	131	1.59
287–293	33.132	71	2.14

**Fig 2:**
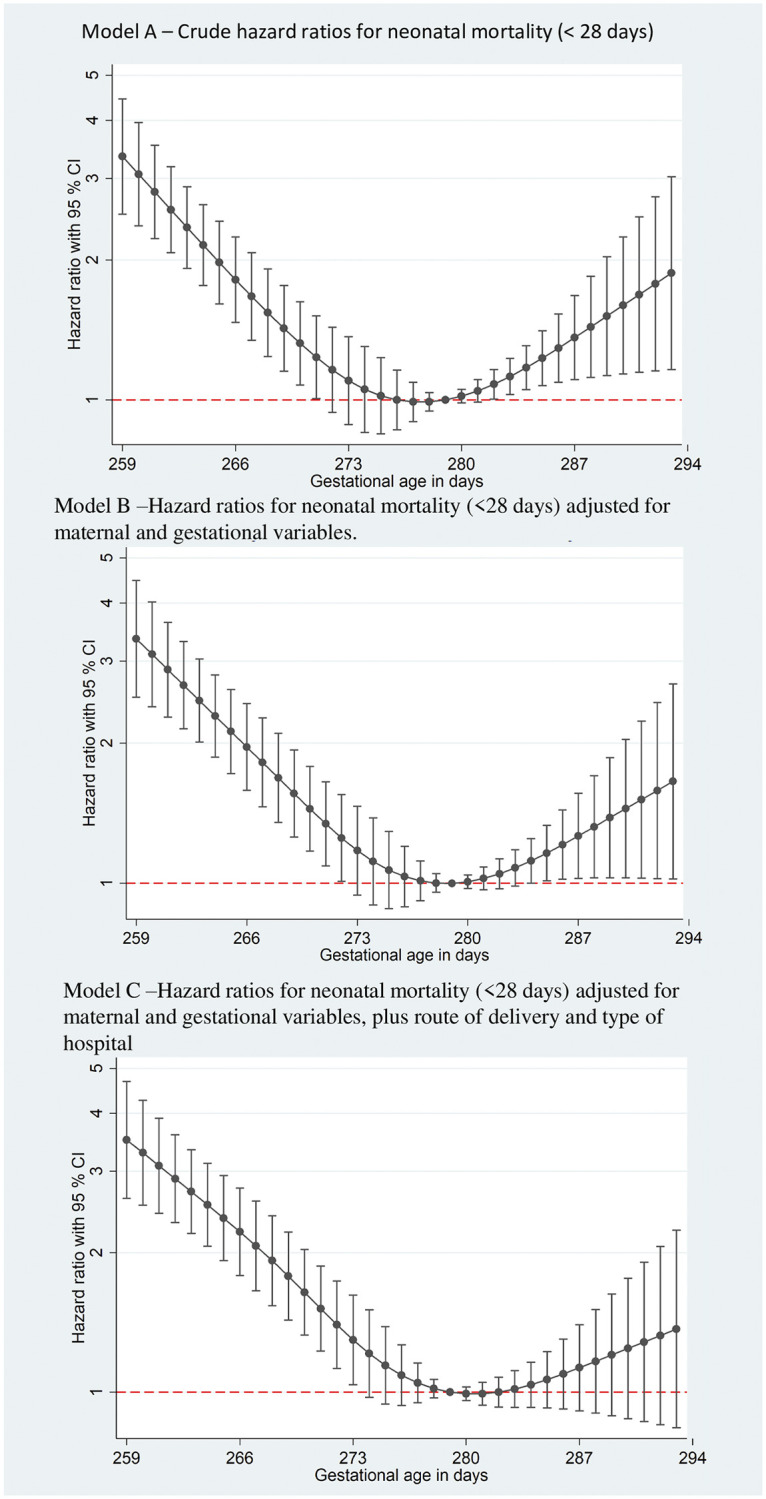
Hazard ratios (HRs) for neonatal mortality (< 28 days of life). (A) Crude hazard ratios. (B) Hazard ratios adjusted for maternal variables (age, skin color, education, living with a partner, parity) and number of antenatal care visits. (C) Hazard ratios adjusted for maternal variables, number of antenatal visits, type of birth and type of hospital.

All NM models had U-shaped curves (Fig 2). In the crude model (Fig 2A), the risk of NM was higher in early-term infants (HR 3.34, 95% CI 2.51 to 4.45 on day 259); the risk decreased as GA approached full term and increased starting at 279 days until the end of the late-term period. In the adjusted model (Fig 2B), we observed a similar pattern, but in Fig 2C, the increase in the late-term period was smaller and not significant (details in S1 File).

**Fig 3:**
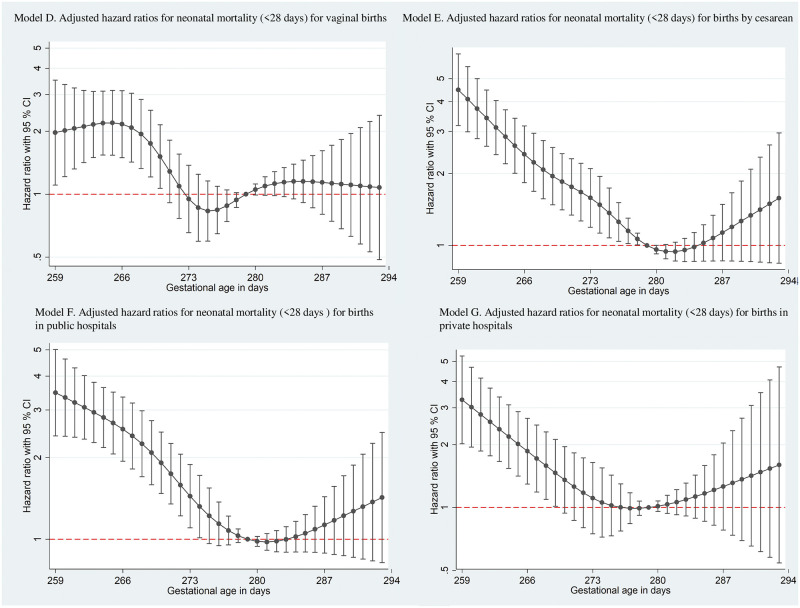
Hazard ratios (HRs) for neonatal mortality adjusted for maternal variables and number of antenatal care visits. Models (D) and (E) stratified according to type of birth. Models (F) and (G) stratified according to type of hospital.

In stratified analyses according to type of birth (Fig 3D and 3E), both had a higher risk of NM for early-term infants, but cesarean births had twice the risk of vaginal delivery in the beginning of these periods, reaching an HR of 4.49 (95% CI 2.51 3.18 to 6.35). In Model E (cesarean), the risk remained significantly high until 278 days, returning to positive values again from 285 days. However, the same was not seen for babies born vaginally, who had nonsignificant protective values between 273 and 278 days.

In the analyses according to the type of hospital (Fig 3F and 3G), we observed the same pattern: the risk of NM was progressively and significantly higher at lower GA in all hospitals. The risk decreased until close to 279 days and rose again.

## Discussion

In the city of São Paulo, babies born in the first days of the term period had a higher risk of NM in all models. This risk decreased for infants born closest to the median GA and increased again afterward. This U-shaped curve suggests that babies born closest to the probable due date (280 days) have the lowest risk of NM.

In all the models, the curves were U-shaped, with higher NM risks at the extremes of the term period. The risk of NM was significantly higher for early-term infants in the three models. The risk of NM was significantly higher for late-term infants only in the crude model, losing its significance in the models that adjusted for maternal characteristics, number of antenatal care visits, and type of birth.

Our results using adjusted models were similar to those reported by Wu et al. [5] in a study involving Danish infants born at 37 completed weeks or later. The authors reported that NM also decreased as GA increased for full-term infants, reaching a risk close to 1 or below starting at 270 days, remaining at this level until the end of the term period. In our dataset, risk inversion occurred one week later, i.e., at 277 days. A similar pattern of increased NM risk in early-term compared to full-term births was reported in a Dutch cohort in 2010–2014 [11]. While all studies showed a higher risk of NM for early-term infants, infants born in Sao Paulo city at full term and late term have a higher risk of NM. Another Brazilian study conducted in the city of Pelotas also reported similar findings [3].

The decrease in the risk of NM in later-term infants in our adjusted models (B and C) showed the effect of covariates, especially type of birth and type of hospital, on the risk of NM in infants born after their due date. This effect was more evident in the stratified models. For early-term infants, vaginal birth (Model D) seemed to be safer than birth by CS (Model E). For example, for babies born at 259 days, the risk of NM for infants born by cesarean section was 3.75 (95% CI 2.87 to 4.90) versus 2.69 (95% CI 1.72 to 4.22) for those born vaginally. The lower risk of NM for babies born vaginally was constant until near the estimated date of birth; this should alert clinicians to avoid CSs before this date. To reduce perinatal risks, several specialist associations, including the American College of Obstetrics and Gynecology, recommend delaying elective CS until 39 weeks of pregnancy [12]. In Brazil, the national medical council issued a statement in 2016 recommending that maternal requests for elective CSs should not be performed before 39 completed weeks of pregnancy [13]. Both models stratified per type of birth indicated protection against NM for babies born after 277 days of pregnancy. Although the HRs between 278–282 days were very similar, the protective effect observed in Model E suggested that cesareans are associated with a lower risk of NM for babies born during this 5-day time period, supporting the recommendation to wait the full term before scheduling the surgery.

Our results indicated a progressive, albeit nonsignificant, increase in the risk of NM in babies born vaginally after 284 days of gestation. In Brazil, there is a high incidence of interventions in women who have a vaginal birth, including artificial rupture of membranes, oxytocin to induce or augment labor, and fundal pressure in the second stage, which are often associated with iatrogenic adverse events [14]. According to Diniz [15], Brazil is experiencing a perinatal paradox with the “worst of two worlds: morbidity and mortality due to lack of appropriate technology, and morbidity and mortality due to excessive use of inappropriate technology”.

The type of hospital had a differential effect on NM in different term periods. While the risk of NM was higher for early-term infants in both types of hospitals (Models F and G), the risk of NM for babies born full term (278–283 days) was lower in public hospitals. This protective effect was not found for babies born in private hospitals.

The median GA at birth in the São Paulo city cohort was 274 days, which was lower than the average GA at birth of 280 days in most international studies [5]. However, our findings were similar to the results of earlier studies in the same city. Both Raspantini et al. [16] and Diniz et al. [10] reported a leftward shift in the median GA at birth in the city of São Paulo, which was more evident for babies born by cesarean section in private (38 weeks) than in public (39 weeks) hospitals. We also found this leftward shift in the GA curve of babies born by cesarean section and in private hospitals, and in our study, both variables were associated with an increased risk of NM for early-term infants.

The high number of neonatal deaths in infants born at term signals the need to improve the quality of care offered to pregnant women in the antenatal period and during birth, since many of these deaths are potentially preventable [17]. One of the main causes of neonatal death in term infants is asphyxia, which is directly related to intrapartum care. This is paradoxical in Brazil, a country with universal health coverage where almost all births occur in hospitals and are managed by licensed health care professionals [15, 17]. To understand the biological plausibility of neonatal deaths in infants born at term, we must take into account many factors, including the interaction between GA and intrapartum interventions, which can have both positive and negative (iatrogenic) effects. The individual variability in the maturity of organic systems between fetuses at the same GA reinforces the need to respect physiology and wait for the spontaneous onset of labor, which could also prevent neonatal deaths associated with obstetric management decisions.

We acknowledge several limitations, such as the lack of data on maternal morbidity that could affect NM, information that is not available in the SINASC database. The lack of data on sonographic GA is another study limitation because of the possible discrepancies in GA estimated by LMP. The lack of information on GA in days in over half of the original sample is an additional limitation. However, a sensitivity analysis did not find significant differences between GA in days between the study population and a sample of the complete original dataset with GA in weeks. This lack of information on GA in days in a large proportion of birth certificates, the main source of information for the SINASC database, indicates the need to improve and standardize how these forms are filled in São Paulo city hospitals.

A strong point of the study was that we used a cohort of live births in the largest city in Brazil, which also has one of the best datasets on births in the country. Additionally, to the best of our knowledge, this is the first Brazilian study to use granular data for GA in days, available in the SINASC database, to analyze the complex problem of NM in a setting where all women who give birth have access to health care.

## Conclusions

In all models, the risk of NM was significantly higher for early-term babies, decreasing until approximately 280 days of gestation, with daily differences. However, contrary to some results of studies conducted in other countries, we found in some models an increased risk of NM for late-term babies. The main hypothesis for this finding is that pregnancies that progress to the late-term period in São Paulo receive more potentially iatrogenic interventions (e.g., induction, augmentation, fundal pressure in the second stage), which could increase the risk of NM in these cases.

The results of this study, from the largest city in South America, can help to understand the role of “potential pregnancy days lost” (including both the effect of fetal immaturity and of interventions in provider-initiated births) in the causes of neonatal mortality, better informing public health policies on maternal and child health. To the best of our knowledge, this is the first Brazilian study on GA in days using a national live births database. Analyses using GA in days and other factors associated with NM at term can help to generate an important discussion on changes in the way data are collected in birth and mortality information systems, showing more clearly the effect of each day on main neonatal outcomes. In this way, this study has the potential to impact health policies across Brazil as well as the health care of women and babies.

## Supporting information

S1 Table Crude and adjusted hazard ratios for neonatal mortality, São Paulo/SP, Brazil. *Adjusted for maternal variables (age, skin color, education, living with a partner, parity), number of antenatal care visits, type of birth and type of hospital.
(DOCX)

S1 File Hazard ratios and confidence intervals (95%) for each day in models A-G. (DOCX)
