# Supplementary material for: Neonatal mortality by gestational age in days in infants born at term: A cohort study in Sao Paulo city, Brazil
Source: PLoS One. 2022 Nov 21;17(11):e0277833. doi: 10.1371/journal.pone.0277833 (PMC9678289; doi:10.1371/journal.pone.0277833)
Supplement: S1 Table — *Adjusted for maternal variables (age, skin color, education, living with a partner, parity), number of antenatal care visits, type of birth and type of hospital. (DOCX) [file pone.0277833.s001.docx]

**S1 Table - Crude and adjusted hazard ratios for neonatal mortality, São Paulo/SP, Brazil.**

| Week | Crude HR | Ajusted* HR |
| --- | --- | --- |
| 37 | 2.36 (1.84-3.03) | 2.91 (2.25-3.75) |
| 38 | 1.38 (1.09-1.76) | 1.87 (1.46-2.39) |
| 39 | 1.01 (0.78-1.30) | 1.21 (0.94-1.56) |
| 40 | 1.00 |  |
| 41 | 1.51 (1.09-2.07) | 1.29 (0.93-1.78) |

* adjusted for maternal variables (age, skin color, education, living with a partner, parity), number of antenatal care visits, type of birth and type of hospital.
