# Supplementary material for: Neonatal mortality by gestational age in days in infants born at term: A cohort study in Sao Paulo city, Brazil
Source: PLoS One. 2022 Nov 21;17(11):e0277833. doi: 10.1371/journal.pone.0277833 (PMC9678289; doi:10.1371/journal.pone.0277833)
Supplement: S1 File — (DOCX) [file pone.0277833.s002.docx]

S1 File – Hazard Ratios and confidence intervals (95%) for each day in models A-G.

**Model A: Crude hazard ratios for neonatal mortality, São Paulo/SP, Brazil**

GA HR (95% CI)

259 3.34 (2.51-4.45)

260 3.06 (2.37-3.95)

261 2.80 (2.22-3.53)

262 2.57 (2.08-3.18)

263 2.35 (1.92-2.88)

264 2.15 (1.76-2.63)

265 1.98 (1.61-2.43)

266 1.81 (1.47-2.24)

267 1.67 (1.34-2.07)

268 1.54 (1.24-1.91)

269 1.43 (1.15-1.76)

270 1.32 (1.08-1.63)

271 1.24 (1.01-1.52)

272 1.16 (0.94-1.43)

273 1.10 (0.89-1.37)

274 1.05 (0.85-1.30)

275 1.02 (0.84-1.23)

276 1.00 (0.86-1.16)

277 0.99 (0.90-1.09)

278 0.99 (0.95-1.04)

279 1.00 (1.00-1.00)

280 1.02 (0.99-1.05)

281 1.05 (0.99-1.11)

282 1.08 (1.00-1.16)

283 1.12 (1.03-1.23)

284 1.17 (1.05-1.31)

285 1.23 (1.07-1.41)

286 1.29 (1.09-1.53)

287 1.36 (1.11-1.68)

288 1.44 (1.12-1.84)

289 1.52 (1.13-2.03)

290 1.60 (1.14-2.24)

291 1.69 (1.15-2.48)

292 1.78 (1.15-2.74)

293 1.87 (1.16-3.02)

**Model B: Hazard ratios for neonatal mortality, adjusted for maternal variables (age, skin color, education, living with a partner, parity) and number of antenatal care visits São Paulo/SP, Brazil**

GA HR (95% CI)

259 3.35 (2.51-4.47)

260 3.10 (2.40-4.02)

261 2.88 (2.28-3.64)

262 2.66 (2.15-3.31)

263 2.47 (2.01-3.03)

264 2.29 (1.87-2.80)

265 2.12 (1.72-2.61)

266 1.96 (1.58-2.43)

267 1.82 (1.46-2.26)

268 1.68 (1.35-2.10)

269 1.56 (1.26-1.93)

270 1.44 (1.17-1.78)

271 1.34 (1.09-1.65)

272 1.25 (1.01-1.55)

273 1.18 (0.94-1.46)

274 1.11 (0.90-1.38)

275 1.07 (0.88-1.29)

276 1.03 (0.89-1.20)

277 1.01 (0.92-1.12)

278 1.00 (0.96-1.05)

279 1.00 (1.00-1.00)

280 1.01 (0.97-1.04)

281 1.02 (0.97-1.08)

282 1.05 (0.97-1.13)

283 1.08 (0.99-1.18)

284 1.12 (1.00-1.25)

285 1.16 (1.01-1.33)

286 1.21 (1.02-1.44)

287 1.26 (1.02-1.56)

288 1.32 (1.03-1.70)

289 1.38 (1.03-1.86)

290 1.45 (1.03-2.04)

291 1.51 (1.03-2.23)

292 1.58 (1.02-2.45)

293 1.66 (1.02-2.68)

**Model C: Hazard ratios for neonatal mortality, adjusted for maternal variables, antenatal visits, type of birth and type of hospital, for neonatal mortality, São Paulo/SP, Brazil**

GA HR (95% CI)

259 3.51 (2.62-4.69)

260 3.29 (2.53-4.27)

261 3.08 (2.43-3.90)

262 2.89 (2.32-3.59)

263 2.71 (2.20-3.34)

264 2.54 (2.07-3.12)

265 2.38 (1.92-2.93)

266 2.22 (1.79-2.76)

267 2.07 (1.66-2.59)

268 1.92 (1.54-2.40)

269 1.78 (1.43-2.22)

270 1.64 (1.33-2.03)

271 1.51 (1.23-1.87)

272 1.40 (1.13-1.74)

273 1.30 (1.04-1.62)

274 1.21 (0.97-1.51)

275 1.14 (0.94-1.38)

276 1.09 (0.94-1.27)

277 1.05 (0.95-1.16)

278 1.02 (0.97-1.07)

279 1.00 (1.00-1.00)

280 0.99 (0.96-1.03)

281 0.99 (0.94-1.05)

282 1.00 (0.93-1.08)

283 1.02 (0.93-1.11)

284 1.04 (0.93-1.16)

285 1.06 (0.93-1.22)

286 1.09 (0.92-1.30)

287 1.13 (0.91-1.40)

288 1.17 (0.90-1.51)

289 1.20 (0.89-1.63)

290 1.24 (0.88-1.76)

291 1.28 (0.86-1.91)

292 1.33 (0.85-2.06)

293 1.37 (0.84-2.24)

**Stratified models**

**Model D. Hazard ratios for neonatal mortality stratified by vaginal births, adjusted for maternal variables, antenatal visits, and type of hospital, São Paulo/SP, Brazil.**

GA HR (95% CI)

259 1.97 (1.11-3.52)

260 2.02 (1.21-3.35)

261 2.06 (1.32-3.22)

262 2.11 (1.42-3.14)

263 2.16 (1.50-3.10)

264 2.19 (1.54-3.11)

265 2.20 (1.54-3.14)

266 2.16 (1.50-3.13)

267 2.08 (1.42-3.04)

268 1.94 (1.33-2.84)

269 1.75 (1.21-2.52)

270 1.51 (1.07-2.15)

271 1.29 (0.91-1.81)

272 1.09 (0.77-1.56)

273 0.95 (0.66-1.37)

274 0.86 (0.60-1.25)

275 0.83 (0.60-1.16)

276 0.84 (0.65-1.09)

277 0.88 (0.74-1.05)

278 0.94 (0.86-1.02)

279 1.00 (1.00-1.00)

280 1.05 (0.99-1.12)

281 1.09 (0.99-1.21)

282 1.12 (0.98-1.28)

283 1.14 (0.97-1.34)

284 1.15 (0.95-1.39)

285 1.15 (0.91-1.46)

286 1.15 (0.86-1.53)

287 1.14 (0.80-1.62)

288 1.13 (0.74-1.71)

289 1.12 (0.68-1.83)

290 1.11 (0.63-1.95)

291 1.10 (0.58-2.08)

292 1.09 (0.53-2.23)

293 1.08 (0.49-2.38)

**Model E. Hazard ratios for neonatal mortality stratified by births by cesarean, adjusted for maternal variables, antenatal visits and type of hospital, for neonatal mortality, São Paulo/SP, Brazil.**

GA HR (95% CI)

259 4.49 (3.18-6.35)

260 4.10 (2.99-5.62)

261 3.74 (2.80-5.00)

262 3.42 (2.61-4.48)

263 3.12 (2.40-4.05)

264 2.85 (2.20-3.70)

265 2.62 (2.01-3.41)

266 2.41 (1.83-3.17)

267 2.23 (1.69-2.95)

268 2.08 (1.57-2.75)

269 1.95 (1.48-2.57)

270 1.85 (1.41-2.43)

271 1.76 (1.34-2.31)

272 1.67 (1.27-2.21)

273 1.58 (1.19-2.10)

274 1.48 (1.13-1.95)

275 1.37 (1.08-1.74)

276 1.25 (1.04-1.51)

277 1.15 (1.02-1.30)

278 1.06 (1.01-1.13)

279 1.00 (1.00-1.00)

280 0.96 (0.92-1.00)

281 0.94 (0.88-1.01)

282 0.94 (0.86-1.03)

283 0.96 (0.86-1.07)

284 0.98 (0.86-1.13)

285 1.02 (0.86-1.22)

286 1.07 (0.86-1.34)

287 1.13 (0.86-1.49)

288 1.20 (0.86-1.66)

289 1.26 (0.86-1.86)

290 1.34 (0.86-2.09)

291 1.41 (0.85-2.35)

292 1.49 (0.85-2.63)

293 1.58 (0.84-2.96)

**Model F. Hazard ratios for neonatal mortality stratified by births in public hospitals, adjusted for maternal variables, antenatal visits and type of birth, São Paulo/SP, Brazil.**

GA HR (95% CI)

259 3.47 (2.41-5.01)

260 3.33 (2.40-4.62)

261 3.20 (2.38-4.29)

262 3.06 (2.34-4.01)

263 2.94 (2.27-3.80)

264 2.81 (2.18-3.63)

265 2.68 (2.06-3.49)

266 2.55 (1.94-3.35)

267 2.41 (1.82-3.18)

268 2.25 (1.70-2.98)

269 2.09 (1.59-2.74)

270 1.92 (1.47-2.49)

271 1.74 (1.35-2.26)

272 1.59 (1.22-2.06)

273 1.44 (1.10-1.89)

274 1.32 (1.01-1.72)

275 1.22 (0.96-1.55)

276 1.14 (0.95-1.37)

277 1.08 (0.95-1.22)

278 1.03 (0.97-1.09)

279 1.00 (1.00-1.00)

280 0.98 (0.94-1.02)

281 0.98 (0.91-1.05)

282 0.98 (0.90-1.08)

283 1.00 (0.90-1.11)

284 1.02 (0.90-1.16)

285 1.05 (0.90-1.23)

286 1.09 (0.90-1.32)

287 1.13 (0.89-1.44)

288 1.17 (0.88-1.57)

289 1.22 (0.87-1.72)

290 1.27 (0.86-1.88)

291 1.32 (0.85-2.06)

292 1.37 (0.83-2.26)

293 1.43 (0.82-2.48)

**Model G. Hazard ratios for neonatal mortality stratified by births in private hospitals, adjusted for maternal variables, antenatal visits and type of birth, São Paulo/SP, Brazil.**

GA HR (95% CI)

259 3.27 (2.02-5.31)

260 3.02 (1.95-4.69)

261 2.79 (1.86-4.16)

262 2.57 (1.77-3.73)

263 2.37 (1.66-3.38)

264 2.18 (1.54-3.10)

265 2.02 (1.41-2.87)

266 1.86 (1.29-2.67)

267 1.72 (1.19-2.48)

268 1.59 (1.09-2.30)

269 1.47 (1.01-2.12)

270 1.36 (0.94-1.95)

271 1.26 (0.87-1.83)

272 1.18 (0.80-1.73)

273 1.11 (0.75-1.64)

274 1.06 (0.72-1.55)

275 1.02 (0.73-1.43)

276 1.00 (0.77-1.30)

277 0.99 (0.84-1.18)

278 0.99 (0.92-1.07)

279 1.00 (1.00-1.00)

280 1.01 (0.96-1.07)

281 1.03 (0.94-1.14)

282 1.06 (0.92-1.22)

283 1.09 (0.91-1.31)

284 1.13 (0.89-1.43)

285 1.17 (0.86-1.59)

286 1.21 (0.82-1.79)

287 1.26 (0.78-2.04)

288 1.31 (0.74-2.33)

289 1.36 (0.70-2.68)

290 1.42 (0.65-3.08)

291 1.48 (0.62-3.54)

292 1.54 (0.58-4.08)

293 1.60 (0.54-4.70)

.
